# Adherence to antiretroviral therapy and the associated factors among people living with HIV/AIDS in Northern Peru: a cross-sectional study

**DOI:** 10.1186/s12981-019-0238-y

**Published:** 2019-08-28

**Authors:** Juan M. Leyva-Moral, Blanca K. Loayza-Enriquez, Patrick A. Palmieri, Genesis M. Guevara-Vasquez, Ursula E. Elias-Bravo, Joan E. Edwards, María Feijoo-Cid, Lucy Y. Davila-Olano, Juan R. Rodriguez-Llanos, Franco E. Leon-Jimenez

**Affiliations:** 1 Departament d’Infermeria, Facultat de Medicina, Universitat Autonoma de Barcelona, Avda. Can Domenech, Building M. Office M3/211, Bellaterra (Cerdanyola del Vallès), 08193 Barcelona, Spain; 2 Center for Health Sciences Research, Universidad María Auxiliadora, Av. Canto Bello 431, San Juan de Lurigancho, Lima, Lima 15408 Peru; 3 Grupo de Investigación Enfermera en Vulnerabilidad y Salud (GRIVIS), Universitat Autonoma de Barcelona, Avda. Can Domenech, Building M. Office M3/211, 08193 Bellaterra (Cerdanyola del Vallès), 08193 Barcelona, Spain; 4 Department of Research, Hospital Regional Lambayecue, Pro. Augusto B. Leguia Nro. 100 (Esquina con Av. Progreso N. 110-120), Chiclayo, Lambayeque 14101 Peru; 5 School of Nursing, Universidad Nacional Pedro Ruiz Gallo, Av. Juan XXIII 391, Lambayeque, Chiclayo 14013 Peru; 6 College of Health Sciences, Universidad Norbert Wiener, Av. Arequipa 444, Lima, Lima 15046 Peru; 7 E​vidence-​B​ased ​H​ealth ​C​are​ South America: A Joanna Briggs Institute Affiliated Group, Universidad María Auxiliadora, Av. Canto Bello 431, San Juan de Lurigancho, Lima, Lima 15408 Peru; 8 Doctor of Health Sciences Program, College of Graduate Health Studies, A. T. Still University, 800 West Jefferson Street, Kirksville, MO 63501 USA; 9 Coordinator HIV/AIDS Unit, Department of Nursing, Hospital Regional Lambayeque, Pro. Augusto B. Leguía Nro. 100 (Esquina con Av. Progreso N. 110-120), Chiclayo, Lambayeque 14101 Peru; 10 Center for Global Nursing, Texas Woman’s University, 6700 Fannin St, Houston, TX 77030 USA; 11 Midwife, Department of Obstetrics, Hospital Regional Lambayeque, Pro. Augusto B. Leguia Nro. 100 (Esquina con Av. Progreso N. 110-120), Chiclayo, Lambayeque 14101 Peru; 12 Department of Medicine, Hospital Regional Lambayeque, Pro. Augusto B. Leguia Nro. 100 (Esquina con Av. Progreso N. 110-120), Chiclayo, Lambayeque 14101 Peru; 13 School of Medicine, Universidad Santo Toribio Mogrovejo, Av. San Josemaría Escriva de Balaguer 855, Chiclayo, Lambayeque 14101 Peru; 14 Department of Medicine, Hospital Regional Lambayeque, Lambayeque, Pro. Augusto B. Leguia Nro. 100 (Esquina con Av. Progreso N. 110-120), Chiclayo, Lambayeque 14101 Peru

**Keywords:** HIV: Human Immunodeficiency Virus, AIDS: Acquired Immune Deficiency Syndrome, ART: Antiretroviral therapy, Adherence, SMAQ: Simplified Medication Adherence Questionnaire, Peru, PLHIV: People living with HIV, Compliance

## Abstract

**Background:**

There are approximately 72,000 people living with HIV/AIDS (PLHIV) in Peru. Non-adherence to antiretroviral therapy (ART) is the most important factor for therapeutic failure and the development of resistance. Peru has achieved moderate progress in meeting the 90-90-90 targets, but only 60% of PLHIV receiving ART are virally suppressed. The purpose of this study was to understand ART adherence in the Peruvian context, including developing sociodemographic and clinical profiles, evaluating the clinical management strategies, and analyzing the relationships between the variables and adherence of PLHIV managed at a regional HIV clinic in Lambayeque Province (Northern Peru).

**Methods:**

This was a cross-sectional study with 180 PLHIV adults, non-randomly but consecutively selected with self-reported ART compliance (78.2% of the eligible population). The PLHIV profile (PLHIV-Pro) and the Simplified Medication Adherence Questionnaire (SMAQ) were used to collect sociodemographic information, clinical variables, and data specific to ART adherence. Descriptive analysis of sociodemographic and clinical characteristics was performed. Bivariate analysis was performed with the Mann–Whitney test, Chi square test, and Yates correction.

**Results:**

The 180 PLHIV sample included 78.9% men, 49.4% heterosexual, 45% with a detectable HIV-1 viral load less than 40 copies/ml, 58.3% not consistently adherent, and only 26.1% receiving Tenofovir + Lamivudine + Efavirenz. Risk factors significant for non-adherence included concurrent tuberculosis, discomfort with the ART regime, and previous pauses in ART. Multivariate analysis of nested models indicated having children is a protector factor for adherence.

**Conclusions:**

Self-reported adherence appeared to be low and the use of first-line therapy is not being prescribed homogeneously. Factors associated with nonadherence are both medical and behavioral, such as having tuberculosis, pausing ART, or experiencing discomfort with ART. The Peruvian government needs to update national technical standards, monitor medication availability, and provide education to health care professionals in alignment with evidence-based guidelines and international recommendations. Instruments to measure adherence need to be developed and evaluated for use in Latin America.

## Introduction

At the end of 2017, there were approximately 2 million [1.5–2.3 million] people living with HIV (PLHIV) in Latin America [1, 2]. In Peru, there are approximately 72,000 [55,000–94,000] PLHIV; however, an estimated 16,250 of these people are unaware of their infection [3]. HIV accounts for 1.8% of the total disease burden and 1.9% of the total annual [2.200] deaths in Peru [4]. Each year, an additional 2700 HIV infections are diagnosed, with 97% related to sexual transmission and men representing 8 of 10 cases [3]. Men who have sex with men (MSM) and transgender women (TW) in Peru are disproportionately impacted by HIV [5], with a prevalence as high as 12.4% for MSM and 30% for TW compared to less than 0.23% for the general population [6, 7]. In addition, MSM and TW account for about 60% of new infections [8]. However, the majority of the HIV literature for these populations in Peru is based on international surveillance or small observational studies focused on the Lima Metropolitan region [7–12]. As such, the number of MSM and TW impacted by HIV/AIDS is largely unknown.

The HIV prevalence worldwide is 1.2%, with the largest prevalence (9.0%) reported in sub-Saharan Africa [13, 14]. The HIV prevalence has incrementally increased from 0.2% to 0.9% in Latin America [15, 16] while the prevalence in Peru remains between 0.4% to 0.5% [17]. From 2010 to 2015; however, new HIV infections in Peru increased by 24% and AIDS-related deaths increased by 14% [18], yet across the region there was only a 3% increase [19]. This trend is attributed to already infected people being diagnosed with increased screenings, problems with late diagnosis, increased heterosexual diagnosis, and HIV + people living longer with ART [20].

Although HIV can be effectively managed with ART as a chronic condition [21] rather than a terminal illness [22], the call by the Joint United Nations Programme on HIV/AIDS (UNAIDS) to enhance ART access in developing economies, such as Peru, was necessary to move governments into action [23]. The Peruvian Ministry of Health, or Ministerio de Salud del Perú (MINSA), directs HIV care through a series of national regulations and guidelines [24–27]. This direction resulted in HIV testing with pretest counseling and signed informed consents except for pregnancy, and blood and organ donation [25]. HIV testing is only free for people with public health insurance [28].

Since 2004, ART has been offered without cost in all regions of Peru [29] through 145 facilities [3]. In 2017, approximately 47,762 PLHIV received ART in Peru, more than double from 2000, resulting in the reduction of AIDS related mortality from 7.3 to 3.9 per 100,000 people from 2000 to 2013 [3, 30]. Overall, Peru has achieved moderate progress in achieving the 90-90-90 targets for 2020 [31] as an estimated 60% (43,000) of PLHIV know their status, 60% (43,000) are receiving ART, and 60% (24,000) in treatment are virally suppressed [5, 32]. Importantly, target measurement is largely based on estimates as there is not a national monitoring system [33].

### Objective

Despite the ART scale-up in Peru, including more resources dedicated to HIV management in an economically constrained health sector, surprisingly few studies focused on ART adherence have been published from Peru [11, 34–40] with none conducted outside the Lima Metropolitan region. Yet, ART adherence is problematic as the facilitators and barriers in Peru are not well understood [34]. The lack of reliable data from most Latin American countries regarding diagnosis, management, and outcomes makes it difficult to implement targeted interventions [1]. As such, the purpose of this study was to understand the current state of ART adherence for PLHIV managed at the regional HIV clinic located in Northern Peru, (Chiclayo, Lambayeque). The study sought to describe the sociodemographic profile of PLHIV, to determine the relationships between sociodemographic variables and ART adherence, and to identify the clinical management compliance with international evidence-based guidelines and recommendations.

This study contributes to the limited South American knowledge about ART adherence outside major metropolitan areas and fills a gap in the Peruvian literature about ART adherence in areas outside of Lima [41]. As there are neither regional nor national registries in Peru specific to ART adherence, data for this study was manually collected through chart reviews and participant interviews. This study is the first reliable source of data specific to PLHIV living in the Peruvian provinces and ART adherence.

## Methods

### Study design and population

This was an observational study with a cross-sectional design [42]. Since the proportion of the ART adherent population is unknown, a non-probabilistic consecutive sampling strategy [43, 44] was implemented in the fall of 2017. From the 230 adult HIV patients managed with ART at the regional HIV clinic in Lambayeque, 180 (78.3%) agreed to participate in this study. The study was approved by the regional health system ethics committee (Protocol 0223-024-16). All participants signed an informed consent prior to entering the study. In addition to the consent document, researchers explained the purpose of the study, including the objectives, risks, benefits, and other ethical aspects. Participants received no financial compensation and they were able to voluntarily leave the study at any time.

### Instruments and measures

The data were collected using two instruments: A self-developed PLHIV profile (PLHIV-Pro) and the Simplified Medication Adherence Questionnaire (SMAQ). The 36-item closed-ended PLHIV-Pro was developed from the available evidence specific to sociodemographic and clinical variables associated with ART adherence and engagement in clinical management. The profile was validated through an external expert review (two nurses and two physicians specialized in HIV care, one patient, and one clinical professor with HIV management experience) and a pilot test was completed with 10 patients to evaluate item comprehension, cultural applicability, and social acceptance. The Simplified Medication Adherence Questionnaire (SMAQ) has 6-tiems, reported to be reliable and valid with a 0.75 α-Cronbach, 82% general inter-observer agreement, 72% sensitivity, and 91% specificity [45]. The instrument was piloted with 10 patients; these results were excluded from analysis. The time to complete the instruments was 10 to 15 min. The two instruments were administered by four researchers (nurses and midwives) to participants following their scheduled health consultation in a private examination room. To avoid bias, data available in the medical record, such as comorbidities, opportunistic infections, viral load, CD4 levels, place of diagnosis, and year of diagnosis, were verified with participant permission.

### Data analysis

Data were analyzed using SPSS v22.0 [46] and EPIDAT v3.1 [47] statistical software packages. First, Kolmogorov–Smirnov normality tests were performed to recognize non-parametric variables [48]. Then, descriptive analysis was used for the qualitative variables, including frequency distribution, and central tendency and dispersion measures were used for the quantitative variables [49]. Next, associations were explore between the participant characteristics and their ART adherence with the Mann–Whitney test [50]. In addition, bivariate analysis of qualitative variables was completed using the Chi square test, Yates correction, and linear tendency (for ordinal variables), with prevalence ratios to calculate associations [51]. Finally, multivariate analysis was conducted using a binomial linear model nested type [52] considering those variables with p-values up to 0.20. A significant “p” value lower than 5% and a confidence interval of 95% of the corresponding statistic was acceptable.

### STROBE compliance

This study is reported in compliance with the STROBE (Strengthening The Reporting of OBservational Studies in Epidemiology) statement [53] (von Elm et al., 2007) specific to the minimum reporting requirements as stated in the cross-sectional study checklist [54].

## Results

The study sample included 180 PLHIV, mostly men (80%; 140) with a median age of 30 years (inter-quartile range of 24 to 38.5 years). Almost half of the participants self-identified as heterosexuals (49.4%; 89) and single (67.8%; 122) with no children (63.9%; 115), and (19.4%; 35), living with a partner, including about half of them living with a same sex partner (11.1%; 20). About half the participants were diagnosed with HIV in a hospital (52.8%; 95), with the sexual route the most prevalent cause (46.7%; 84 homosexual). Participants primarily lived in urban areas (75.6%; 136), completed university studies (39.4%, 71), and worked self-employed (40%; 72) with a median family monthly income of 1000 soles, or about $330 (inter-quartile range of 625 to 1500 soles). A summary of the relevant socio-demographic and ART adherence characteristics are provided in Table 1. Also, the complete data are available in the Additional file 1: Table S1.

**Table 1:** Summary of socio-demographic characteristics of PLHIV and ART adherence at the HIV clinic (2016–2017)

Variable	N (%)	Non-adherence/total (%)	PR [CI 95%]	*p* value
Age**	30 [24–38.50]	29 [24–39]		0.646
Sex				
Male	144 (80.0)	81/144 (56.3)		
Female	36 (20.0)	24/36 (66.7)	0.84 [0.64–1.11]	0.257
Sexual orientation				
Homosexual	63 (35.0)	35/63 (55.6)		0.853
Heterosexual	89 (49.4)	53/89 (59.6)	1.07 [0.81–1.42]	
Bisexual	28 (15.6)	17/28 (60.7)	1.09 [0.75–1.58]	
Marital status				
Single	122 (67.8)	68/122 (55.7)		0.778
Living with someone/partner	35 (19.4)	22/35 (62.9)	1.13 [0.84–1.52]	
Married	12 (6.7)	8/12 (66.7)	1.20 [0.78–1.84]	
Divorced/widowed	11 (6.1)	7/11 (63.6)	1.14 [0.71–1.83]	
Number of children				
0	115 (63.9)	60/115 (52.2)		0.029*
1	21 (11.7)	13/21 (61.9)	1.19 [0.81–1.73]	
2	25 (13.9)	19/25 (76)	1.46 [1.10–1.93]	
≥ 3	19 (10.6)	13/19 (68.4)	1.31 [0.92–1.86]	
Monthly family incomes**	1000 [625–1500]	1000 [700–1800]		0.302
ART prescription				
ZDV + 3TC + EFV	62 (34.4)	39/62 (62.9)		0.861
TDF + 3TC + EFV	47 (26.1)	25/47 (53.2)	0.85 [0.61–1.18]	
ABC + 3TC + EFV	26 (14.4)	14/26 (53.8)	0.86 [0.57–1.28]	
TDF + 3TC + ATV + RTV	15 (8.3)	9/15 (60)	0.95 [0.61–1.50]	
Other	30 (16.7)	18/30 (60)	0.95 [0.67–1.35]	
Place of diagnosis				
Hospital	95 (52.8)	60/95 (63.2)		0.035*
Primary Health Center	59 (32.8)	32/59 (54.2)	0.86 [0.65–1.14]	
Private lab or clinic	16 (8.9)	5/16 (31.3)	0.49 [0.24–1.04]	
Ambulatory care	10 (5.6)	8/10 (80)	1.27 [0.90–1.79]	
Viral Load				0.513
> 40	81 (45.0)	51/81 (63)		0.255
≤ 40	99 (55.0)	54/99 (54.5)	1.15 [0.90–1.48]	
Nivel de CD4**	356 [261–527]	362 [264–516]		0.879
≤ 300	66 (36.7)	37/66 (56.1)		0.638
> 300	114 (63.3)	68/114 (59.6)	0.94 [0.72–1.22]	
Years with no ART since diagnosis**	2 [1–5]	2 [1–5]		0.662
≥ 1	45 (25)	26/45 (57.8)		0.930
< 1	135 (75)	79/135 (58.5)	0.99 [0.74–1.32]	
Years living with HIV**	2 [1–3]	2 [1–3]		0.980
≥ 1	141 (78.3)	86/141 (61)		0.169
< 1	39 (21.7)	19/39 (48.7)	1.25 [0.88–1.77]	
Years taking ART**	1 [1, 2]	1 [1, 2]		0.988
≥ 1	122 (67.8)	73/122 (59.8)		0.553
< 1	58 (32.2)	32/58 (55.2)	1.08 [0.82–1.43]	
Opportunistic infection				
Yes	80 (44.4)	43/80 (53.8)		0.265
No	100 (55.6)	62/100 (62)	0.87 [0.67–1.12]	
Pulmonary tuberculosis				
Yes	18 (10.0)	6/18 (33.3)		0.023*
No	162 (90.0)	99/162 (61.1)	0.55 [0.28–1.06]	
Side effects with actual ART prescription				
Yes	92 (51.1)	62/92 (67.4)		0.012*
No	88 (48.9)	43/88 (48.9)	1.38 [1.07–1.78]	
Action taken with side effects				
Ask for medical advice	57 (62.0)	38/57 (66.7)		0.031*
Wait it to stop	28 (30.4)	22/28 (78.6)	1.18 [0.90–1.54]	
Keep taking my ART	4 (4.3)	2/4 (50)	0.75 [0.28–2.03]	
Did not answer	3 (3.3)	0/3 (0)	–	
ART abandoned at anytime				
Yes	83 (46.1)	72/83 (86.7)		0.001*
No	97 (53.9)	33/97 (34)	2.55 [1.91–3.41]	
Length of ART abandon				
> 30 days	15 (18.1)	9/15 (60)		0.001*
10–30 days	16 (19.3)	14/16 (87.5)	1.46 [0.93–2.29]	
1–9 days	52 (62.7)	49/52 (94.2)	1.57 [1.03–2.39]	
Reason for abandonment				
Forgetfulness or neglect when attending a commitment, meeting or work	35 (40.7)	35/35 (100)		0.033*
Bureaucracy to get access to ART	17 (19.8)	13/17 (76.5)	0.76 [0.59–1.00]	
Lack of privacy or feeling better	11 (12.8)	9/11 (81.8)	0.82 [0.62–1.08]	
Fear of side effects	8 (9.3)	6/8 (75)	0.75 [0.50–1.12]	
Others	15 (17.4)	11/15 (73.3)	0.73 [0.54–1.00]	

Zidovudine = ZDV; Lamivudine = 3TC; Efavirenz = EFV; Tenofovir = TDF; Abacavir = ABC; Atazanavir = ATV; Ritonavir = RTV; Lopinavir = LPV; PR = prevalence ratio

* p-value < 0.05 ** Mean [IQR]

The median time living with a diagnosis of HIV was 2 years (IQR 1–3), with most participants (75%; 135) starting ART within 1 year (mean 2 years; IQR 1–5). Only 21.7% (39) of the participants were ART-naive (mean 1 year; IQR 1–2). For the ART, the most frequent drug combination was Zidovudine + Lamivudine + Efavirenz (34.4%; 62). More than half of the participants (55%; 99) had an undetectable HIV-1 viral load (≤ 40 copies/ml), and 63.3% (114) had CD4 monitoring ≥300 cel/μL (mean 356; IQR 261–527 cel/μL).

Many participants (108) reported comorbidities, depression was the most prevalent (42.2%; 76). Among participants diagnosed with a sexually transmitted infection (30%; 54), syphilis was the most frequent (12.8%; 23). Most participants shared their HIV diagnosis with a family member (79.4%; 143), primarily because they wanted emotional support managing the treatment (64.3%; 92). Among those who did not share their HIV diagnosis with their family (19.4%; 35) the main reason was fear of rejection, personal shame, and lack of confidence (32.4%; 12), or not wanting to cause more problems (32.4%; 12). A high percentage of participants reported satisfaction with the care they received from their physician (90%; 162) and higher satisfaction with the nursing care (97.2%; 175).

In terms of ART adherence, 58.3% (105) of the participants were not adherent, with 43.3% (78) sometimes forgetting to take their ART. In this regard, about half stopped their ART regime at some point (46.1%; 83), in most of these cases up to 9 days (62.7%; 52), with the main cause being “forgetfulness or neglect when attending a commitment, meeting, or work” (40.7%; 35). During the last month prior to this study, only 10% (18) of participants forgot their ART doses for 1–2 days, and another 5.6% (10) stopped taking their ART as they felt sick. More than half of the participants had side effects with their current ART regime (51.1%; 92), including heartburn/stomach pain (13.3%; 24) and skin rash (11.7%; 21) as the most frequent. Some participants (22.2%; 40) also acknowledged at least moderate alcohol consumption (Table 2).

**Table 2:** Adherence characteristics of people receiving ART at the HIV Clinic (2016–2017)

Variable	Category	n	%
Adherence ≥ 90%	Yes	75	41.7
	No	105	58.3
Did you ever forget to take your medication?	Yes	78	43.3
	No	99	55.0
	Did not answer	3	1.7
I always take my medication at the indicated time	Yes	130	72.2
	No	46	25.6
	Did not answer	4	2.2
I stop taking my medication when I feel sick	Yes	10	5.6
	No	164	91.1
	Did not answer	6	3.3
I forget to take my medication on the weekends	Yes	8	4.4
	No	166	92.2
	Did not answer	6	3.3
Number of days I forgot to take my medication in the last week	None	156	86.7
	1 day	17	9.4
	2–3 days	2	1.1
	4–5 days	2	1.1
	> 5 days	3	1.7
Number of days I forgot to take my medication in the last month	None	149	82.8
	1–2 days	18	10.0
	3–5 days	4	2.2
	6–10 days	6	3.3
	Did not answer	3	1.7

The bivariate analysis indicated participant discomfort with the treatment regime (p = 0.012; RP = 1.38; IC 95% 1.07–1.78); stopping treatment at any time (p < 0.001; RP = 2.55; IC 95%:1.91–3.41) and stopping treatment for up to 9 days (p = 0.001; RP = 1.57; IC 95%:1.03–2.39) were significant risk factors for non-adherence (Table 3). Other risk factors for non-adherence included concurrent TBC (p = 0.013; RP = 5.19; IC 95%:1.42–18.91), feeling sick during the ART regime (p = 0.012; RP = 2.73; IC 95%:1.24–6.00), and stopping ART at some point (p < 0.001; RP = 17.17; IC 95%:7.19–41). In addition, multivariate analysis of nested models indicated having children could be a significant protective factor for adherence (p = 0.024; RP = 0.25; IC 95%:0.08–0.84).

**Table 3:** Multivariate analysis of sociodemographic characteristics and ART adherence for people managed at the HIV clinic (2016–2017)

Variable	Bivariate		Multivariate	
	RP [IC 95%]	p-valor	RP [IC 95%]	p-valor
Number of children				
None		0.029*		0.024*
1	1.19 [0.81–1.73]		0.25 [0.08–0.84]	
2	1.46 [1.10–1.93]		0.24 [0.07–0.83]	
≥ 3	1.31 [0.92–1.86]		0.21 [0.06–0.77]	
Place of diagnosis				
Hospital		0.045*		
Primary Care Center	0.86 [0.65–1.14]			
Private lab or clinic	0.49 [0.24–1.04]			
Ambulatory care	1.27 [0.90–1.79]			
TBC				
Yes		0.023*		0.013*
No	0.55 [0.28–1.06]		5.19 [1.42–18.91]	
Complaints about the current ART regime				
Yes		0.012*		0.012*
No	1.38 [1.07–1.78]		2.73 [1.24–6.00]	
Action taken when experiencing side effects				
Visit the physician		0.041*		
Nothing. just wait it to stop	1.18 [0.90–1.54]			
Take my ART anyway	0.75 [0.28–2.03]			
Did not answer	–			
ART cessation ever				
Yes		p < 0.001*		p < 0.001*
No	2.55 [1.91–3.41]		17.17 [7.19–41]	
Length of last ART cessation				
> 30 days		0.001*		
10–30 days	1.46 [0.93–2.29]			
1–9 days	1.57 [1.03–2.39]			
Reason for cessation ART				
Forgetfulness or neglect when attending a commitment. meeting or work		0.033*		
Permissions. paperwork for getting access to ART	0.76 [0.59–1.00]			
My own decision. lack of privacy or wanting to feel better	0.82 [0.62–1.08]			
Fear of side effects	0.75 [0.50–1.12]			
Other	0.73 [0.54–1.00]			

## Discussion

The participants in this study were mostly young (24.0 to 38.5 years) men (80%; 144). The socioeconomic data from this study is similar to the national data [18] and data reported by other countries in the region such as Colombia [55], reflecting a globalized reality that HIV adversely impacts young men. Participants in this study were mostly self-identified as heterosexual (49.4%; 89) and homosexual (35%; 63). These figures are similar to the United Kingdom where, in 2011, heterosexual infections accounted for 49% of all adult 5423 diagnoses [56]. Contrarily, in other countries, such as Spain [57] and the United States [58] only about 25% were identified as heterosexual, with the highest number of diagnoses (53.6%) in MSM. The difference in our study could be explained by the pervasive discrimination experienced by Lesbian, Gay, Transgender, and Bisexual people in Latin America [59]. For example, more than 45% of Peruvian adult women surveyed would not buy vegetables from a shopkeeper known to be living with HIV [2]. This type of discrimination might influence participant responses to some of the questions.

### Adequacy of clinical management

Regarding ART clinical management, the World Health Organization [60] states, “ART should be started in all adults living with HIV, regardless of WHO clinical stage and at any CD4 cell count” (p. xxxi). Early ART initiation is associated with decreased new infections [61]. However, the technical standard published by the Peruvian government [27] specify ART based on different criteria (Table 4). In addition, the World Health Organization [60] recommendation, and the Peruvian government [27], indicate the combination of Tenofovir + Lamivudine + Efavirenz is the first choice for ART; however, only 26% of the participants in this study received this combination. Yet, more participants, 34.4% received the less effective alternative treatment [60], Zidovudine + Lamivudine + Efavirenz. During the data collection process for this study, clinicians and participants indicated the recommended ART medications were not routinely available in the region. Despite published information that the WHO recommended ART is widely available in Peru, local HIV experts suggested the provinces routinely lack Lamivudine and/or Tenofovir. This demonstrates the reality reported in literature for Metropolitan Lima may differ from the experiences in the provinces.

**Table 4:** Technical standard (NTS No. 097 MINSA/DGSP-V.02) in Peru for comprehensive care of the adult with Human Immunodeficiency Virus (HIV) infection

Any person with HIV infection who has symptoms related to immunosuppression (clinical stages 2, 3 and 4 of the WHO 2007 classification)
Any person with HIV infection who has a CD4 T cell count ≤ 500 cells/μL, regardless of the presence of symptoms
Any person with HIV infection, regardless of the presence of symptoms and CD4 T cell count, who has any of the following conditions
HIV-related nephropathy
Neuro-cognitive impairment associated with HIV
Non-HIV neoplasms that require chemotherapy or radiation therapy
Coinfection with chronic hepatitis B requiring treatment
Chronic hepatitis C co-infection requiring treatment
HIV-related autoimmune diseases
Gestation
Anyone with acute HIV infection who has symptoms
Serodiscordant couples
Other cases, according to the criteria of the treating physician after consultation and approval of the Expert Committee on Comprehensive Care for Adults with HIV Infection

Also, each PLHIV presenting at the regional clinic is initially evaluated by a psychologist. In the presence of a suspected mental condition, a PLHIV is required to be referred to a psychiatrist for diagnosis and clinical management. Similar to other studies [62–65] most participants in this study had a depression diagnosis. This is the first study to report depression as a problem for PLHIV in Peru. Importantly, the local experts reported the initial psychological evaluations are completed in a timely manner; however, there are limited appointments available for referrals and continued management.

### ART adherence rates

Although the ART adherence rates reported in the literature are wide-ranging and dependent on the socio-cultural context, there needs to be a good assessment tool to measure adherence in Latin American countries. Data reported in a meta-analysis from the United States reported the adherence rate to be 55%, with good adherence deemed to be 80%. When correcting this data with the 90% expectation, as is the case in this study, good adherence descends to 62% [66]. Furthermore, studies [67] with small samples from the region report adherence rates for PLHIV as high as 90%. Yet, older but slightly large studies in Peru [36] identified higher adherence rates only among people with higher education (OR = 0.45. 95% CI 0.27–0.75) and age (OR = 1.05. 95% CI 1.02–1.08). Additional research is necessary to measure ART adherence across Peru.

### Detectable viral load and ART adherence

In an unpublished undergraduate thesis studying PLHIV in Northern Peru, ART adherence was reported to be 30%, with an association identified between physical and mental quality of life and ART adherence (p = 0.03 and 0.04) [68]. Although adherence in this study was higher, almost half of the patients (45%) had a detectable viral load. This result must be considered with caution as patients in Peru learn to obey physicians and interface with the health system with largely passive behaviours. As a result, patients who were not adherent might have decided to respond affirmatively. In addition, the detectable viral load can be partially explained by people beginning ART within the last 6 months. As 32.2% of patients were in their first year of ART, they could still be adapting to the daily medication intake as well as the newness of any side effects. Furthermore, the inadequate incorporation of the WHO recommended ART for most participants could have limited the clinical effectiveness of treatment. The policy opportunity is to develop a regional database to monitor program outcomes, such as ART regimen, ART adherence, and level of viral resistance.

### Patient satisfaction and ART adherence

The results indicate complaints about the ART regimen were a significant factor associated with non-adherence. In the metanalysis by Clay, Nag, Graham, and Narayanan [69], PLHIV taking a single pill were significantly more adherent in comparison to those taking more than one pill at any frequency (odds ratio [OR]: 2.37 [95% CI 1.68, 3.35], p  <  0.001), twice-daily MTR (OR: 2.53 [95% CI 1.13, 5.66], p =  0.02) and the once-daily multiple tablet regimens (OR: 1.81 [95% CI 1.15, 2.84], p  =  0.01). Similarly, Raffi et al. [70] found significantly higher rates of ART adherence (89.6%) among those taking a single tenofovir-emtricitabine-efavirenz therapy, compared to those taking combinations of ART (86.4% > 1 pill once daily; 77.0% > 1 daily intake; P  <  0.0001). As such, the policy implication is for the MINSA to consider the cost to benefit ratio for shifting ART to a one pill per day regimen.

### Tuberculosis comorbidity and ART adherence

No prior evidence has been found reporting Tuberculosis (TB) comorbidity as a risk factor of ART non-adherence. However, research indicates PLHIV have a 20- to 37-fold higher risk of active TB than those without HIV [71]. The association found in this study can be explained by the summative effect of suffering from two chronic conditions, associated with stigma and discrimination, that require clinical management for an entire life. Furthermore, this study found interrupted ART at some point is a risk factor for non-adherence (p < 0.001; RP = 17.17; IC 95%:7.19–41), more significantly for PLHIV with concurrent TB. The literature indicates ART interruptions are common, especially in the presence of other chronic conditions, due to the resulting fatigue and personal attempts to eliminate side effects with treatment [72].

### Study limitations

Despite this being one of the larger studies conducted in Latin America, and the largest in Peru, this study has several limitations. First, the descriptive design with a relatively small sample size, compared to studies outside the region, was used to calculate frequencies and averages, and to perform bivariate exploratory analysis. As such, the design might not show important positive associations. However, the study sample was comprised on almost 80% of the available population in the region, so the results are at least a good approximation of the local reality. Second, access to HIV data, requires locating and reviewing health records in an environment with deficits in storage, limitations in standards, and inconsistencies in the organization of clinical data. This Peruvian reality can lead to errors in data collection or even missed data. Although double-checks were incorporated into this study, the possibility for errors could impact the results. Third, despite the widespread use of the instrument to calculate adherence, there are few studies that establish the positive predictive value; possibly distorting the calculated rates. However, this study included a review of the health record with a comparison to the laboratory tested viral load as a verification process. Fourth, the adherence measurement was cross-sectional; therefore, the data is limited to one moment in time versus the adherence changes over time (this was not a study objective). Fifth, the laboratory values were collected from medical records and referred to the last value recorded within the last 6 months. As such, there could be variations in lab value recorded with any pending, or misplaced, values. Despite these limitations, the resulting data was collected in a systematic and rigorous manner. Finally, despite the consecutive sampling strategy there was a low TW participation in this study. Clinicians report the TW population encounter severe stigma and pervasive discrimination in the provinces that forces them to remain “incognito” in the health system. In retrospect, a proportional quota sampling strategy might have addressed this limitation [73, 74].

## Conclusion

PLHIV managed at the regional clinic in Lambayeque are mainly young men, self-identified as heterosexual, urban residents, with high school education, and lower incomes. Being co-infected with TB, having complaints about the ART regimen, and interrupting ART at some point are risk factors for non-adherence. Given the adherence rates reported in this study, the analytical values observed, and the variations in the ART management from the internationally published evidence-based standards for care of PLHIV, there are two important policy implications. First, the Peruvian national clinical practice guidelines for HIV/AIDS need to be evaluated and compared with contemporary evidence-based clinical practices and existing international standards of care. Second, the clinical practices at the regional level need to be evaluated against the national guidelines to determine the level of ART regimen compliance. These two recommendations translate into the revision of the technical standards to include current international recommendations regarding the initiation of ART and insuring the first-line medications are available to all Peruvians, not only those living in Lima.

There needs to be further research to develop strategies tailored for the Peruvian reality that achieve improvements in empowerment, self-management, and self-care that result in better ART adherence, and indirectly reduce the number of new infections. Furthermore, processes need to be implemented to increase the early diagnoses of HIV in order to improve timely treatment, reduce complications, and insure the cost-effective distribution of limited resources. This implies establishing an interdisciplinary community-based intervention to increase access to diagnostic testing for HIV in ambulatory settings versus the hospital, and to educate physicians about when and how to refer people for HIV testing.

In closing, the experience from this study needs to be translated into the development of a permanent local registry for the province where the regional clinic is located to evaluate therapeutic successes and failures. The registry should use the UNAIDS 90-90-90 targets as the primary outcome measures and then the five segments of the HIV Care Continuum to guide the construction of process measures. This can be accomplished by implementing an evidence-based interdisciplinary program, such as the Adherence Improving Self-Management. Overall, the effectiveness of HIV clinical management and pharmacological interventions need to be evaluated in order to identify the best practices and to disseminate the valuable “lessons learned” that facilitate a more effective scale-up of programs in the region.

## Supplementary information

**Additional file 1: Table S1.** All socio-demographic characteristics of PLHIV and ART adherence at the HIV clinic (2016–2017).

## Data Availability

The majority of data collected and analyzed for this study are included in the published article and the Additional files. The complete datasets for this study are not publicly available due to the possibility for a breach of participant privacy, persons living with HIV/AIDS, in a relatively small population clinically managed at a single hospital location. However, the data will be made available to researchers, upon written request to the corresponding author, with the reason for soliciting the data.
