# Supplementary material for: Adherence to antiretroviral therapy and the associated factors among people living with HIV/AIDS in Northern Peru: a cross-sectional study
Source: AIDS Res Ther. 2019 Aug 28;16:22. doi: 10.1186/s12981-019-0238-y (PMC6714391; doi:10.1186/s12981-019-0238-y)
Supplement: Supplementary file 1 — Additional file 1: Table S1. All socio-demographic characteristics of PLHIV and ART adherence at the HIV clinic (2016–2017). [file 12981_2019_238_MOESM1_ESM.docx]

Table S1. All socio-demographic characteristics of PLHIV and ART adherence at the HIV clinic (2016-2017).

| **Variable** | **N (%)** | **Non- adherence / Total (%)** | | **PR [CI 95%]** | **p-value** |
| --- | --- | --- | --- | --- | --- |
| Age** | 30 [24-38.50] | 29 [24-39] | |  | 0.646 |
| Sex |  |  | |  |  |
| Male | 144 (80.0) | 81/144 (56.3) | |  |  |
| Female | 36 (20.0) | 24/36 (66.7) | | 0.84 [0.64-1.11] | 0.257 |
| Sexual Orientation |  |  | |  |  |
| Homosexual | 63 (35.0) | 35/63 (55.6) | |  | 0.853 |
| Heterosexual | 89 (49.4) | 53/89 (59.6) | | 1.07 [0.81-1.42] |  |
| Bisexual | 28 (15.6) | 17/28 (60.7) | | 1.09 [0.75-1.58] |  |
| Marital Status |  |  | |  |  |
| Single | 122 (67.8) | 68/122 (55.7) | |  | 0.778 |
| Living with someone / partner | 35 (19.4) | 22/35 (62.9) | | 1.13 [0.84-1.52] |  |
| Married | 12 (6.7) | 8/12 (66.7) | | 1.20 [0.78-1.84] |  |
| Divorced / Widowed | 11 (6.1) | 7/11 (63.6) | | 1.14 [0.71-1.83] |  |
| Actual partner |  |  | |  |  |
| Do not have | 108 (60.0) | 60/108 (55.6) | |  | 0.431 |
| Male | 43 (23.9) | 25/43 (58.1) | | 1.05 [0.77-1.42] |  |
| Female | 29 (16.1) | 20/29 (69) | | 1.24 [0.92-1.67] |  |
| Number of children |  |  | |  |  |
| 0 | 115 (63.9) | 60/115 (52.2) | |  | 0.029* |
| 1 | 21 (11.7) | 13/21 (61.9) | | 1.19 [0.81-1.73] |  |
| 2 | 25 (13.9) | 19/25 (76) | | 1.46 [1.10-1.93] |  |
| ≥3 | 19 (10.6) | 13/19 (68.4) | | 1.31 [0.92-1.86] |  |
| Area of residence |  |  | |  |  |
| Urban | 136 (75.6) | 77/136 (56.6) | |  | 0.715 |
| Rural | 33 (18.3) | 21/33 (63.6) | | 1.12 [0.84-1.51] |  |
| Urban – outskirts | 11 (6.1) | 7/11 (63.6) | | 1.12 [0.70-1.80] |  |
| Level of studies |  |  | |  |  |
| Primary | 19 (10.6) | 12/19 (63.2) | |  | 0.364 |
| Secondary | 48 (26.7) | 29/48 (60.4) | | 0.96 [0.63-1.45] |  |
| Superior Technician | 42 (23.3) | 26/42 (61.9) | | 0.98 [0.65-1.49] |  |
| College | 71 (39.4) | 38/71 (53.5) | | 0.85 [0.56-1.27] |  |
| Current occupation |  |  | |  |  |
| Self-employed | 72 (40.0) | 44/72 (61.1) | |  | 0.447 |
| Employed | 29 (16.1) | 15/29 (51.7) | | 0.85 [0.57-1.26] |  |
| Student | 30 (16.7) | 16/30 (53.3) | | 0.87 [0.60-1.28] |  |
| Unemployed | 27 (15) | 18/27 (66.7) | | 1.09 [0.79-1.51] |  |
| Housewife | 13 (7.2) | 9/13 (69.2) | | 1.13 [0.75-1.70] |  |
| Other | 9 (5.0) | 3/9 (33.3) | | 0.55 [0.21-1.40] |  |
| Monthly family incomes** | 1,000 [625-1,500] | 1,000 [700-1,800] | |  | 0.302 |
| ART prescription |  |  | |  |  |
| ZDV+3TC+EFV | 62 (34.4) | 39/62 (62.9) | |  | 0.861 |
| TDF+3TC+EFV | 47 (26.1) | 25/47 (53.2) | | 0.85 [0.61-1.18] |  |
| ABC+3TC+EFV | 26 (14.4) | 14/26 (53.8) | | 0.86 [0.57-1.28] |  |
| TDF+3TC+ATV+RTV | 15 (8.3) | 9/15 (60) | | 0.95 [0.61-1.50] |  |
| Other | 30 (16.7) | 18/30 (60) | | 0.95 [0.67-1.35] |  |
| Place of diagnosis |  |  | |  |  |
| Hospital | 95 (52.8) | 60/95 (63.2) | |  | 0.035* |
| Primary Health Center | 59 (32.8) | 32/59 (54.2) | | 0.86 [0.65-1.14] |  |
| Private lab or clinic | 16 (8.9) | 5/16 (31.3) | | 0.49 [0.24-1.04] |  |
| Ambulatory care | 10 (5.6) | 8/10 (80) | | 1.27 [0.90-1.79] |  |
| Viral Load |  |  | |  | 0.513 |
| >40 | 81 (45.0) | 51/81 (63) | |  | 0.255 |
| ≤40 | 99 (55.0) | 54/99 (54.5) | | 1.15 [0.90-1.48] |  |
| Nivel de CD4** | 356 [261-527] | 362 [264-516] | |  | 0.879 |
| ≤300 | 66 (36.7) | 37/66 (56.1) | |  | 0.638 |
| >300 | 114 (63.3) | 68/114 (59.6) | | 0.94 [0.72-1.22] |  |
| Years with no ART since diagnosis** | 2 [1-5] | 2 [1-5] | |  | 0.662 |
| ≥1 | 45 (25) | 26/45 (57.8) | |  | 0.930 |
| <1 | 135 (75) | 79/135 (58.5) | | 0.99 [0.74-1.32] |  |
| Years living with HIV** | 2 [1-3] | 2 [1-3] | |  | 0.980 |
| ≥1 | 141 (78.3) | 86/141 (61) | |  | 0.169 |
| <1 | 39 (21.7) | 19/39 (48.7) | | 1.25 [0.88-1.77] |  |
| Years taking ART** | 1 [1-2] | 1 [1-2] | |  | 0.988 |
| ≥1 | 122 (67.8) | 73/122 (59.8) | |  | 0.553 |
| <1 | 58 (32.2) | 32/58 (55.2) | | 1.08 [0.82-1.43] |  |
| Transmission Route |  |  | |  |  |
| Sexual homosexual | 84 (46.7) | 47/84 (56) | |  | 0.927 |
| Sexual heterosexual | 64 (35.6) | 39/64 (60.9) | | 1.09 [0.83-1.43] |  |
| Unknown | 30 (16.7) | 18/30 (60) | | 1.07 [0.76-1.52] |  |
| Transfusion | 2 (1.1) | 1/2 (50) | | 0.89 [0.22-3.62] |  |
| Within the last 6 months had sex mainly with… | | | | |  |
| Men | 84 (46.7) | 51/84 (60.7) | |  | 0.105 |
| Women | 37 (20.6) | 24/37 (64.9) | | 1.07 [0.80-1.43] |  |
| Both | 7 (3.9) | 6/7 (85.7) | | 1.41 [1.00-2.00] |  |
| None | 52 (28.9) | 24/52 (46.2) | | 0.76 [0.54-1.07] |  |
| Condom use in the last sexual encounters | | | | | |
| Never/sometimes | 7 (5.3) | 3/7 (42.9) |  | | 0.790 |
| Almost always | 13 (9.8) | 10/13 (76.9) | 1.79 [0.73-4.44] | |  |
| Always | 112 (84.8) | 69/112 (61.6) | 1.44 [0.60-3.42] | |  |
| Comorbidities |  |  |  | |  |
| Si | 108 (60.0) | 67/108 (62) |  | | 0.217 |
| No | 72 (40.0) | 38/72 (52.8) | 1.18 [0.90-1.53] | |  |
| Depression |  |  |  | |  |
| Yes | 76 (42.2) | 46/76 (60.5) |  | | 0.610 |
| No | 104 (57.8) | 59/104 (56.7) | 1.07 [0.83-1.37] | |  |
| Pulmonary disease |  |  |  | |  |
| Yes | 12 (6.7) | 8/12 (66.7) |  | | 0.545 |
| No | 168 (93.3) | 97/168 (57.7) | 1.15 [0.76-1.76] | |  |
| Gastritis |  |  |  | |  |
| Yes | 18 (10.0) | 12/18 (66.7) |  | | 0.450 |
| No | 162 (90.0) | 93/162 (57.4) | 1.16 [0.82-1.65] | |  |
| Anxiety |  |  |  | |  |
| Yes | 9 (5.0) | 5/9 (55.6) |  | | 0.862 |
| No | 171 (95.0) | 100/171 (58.5) | 0.95 [0.52-1.73] | |  |
| Acarosis |  |  |  | |  |
| Yes | 14 (7.8) | 11/14 (78.6) |  | | 0.110 |
| No | 166 (92.2) | 94/166 (56.6) | 1.39 [1.02-1.88] | |  |
| Oportunistic infecion |  |  |  | |  |
| Yes | 80 (44.4) | 43/80 (53.8) |  | | 0.265 |
| No | 100 (55.6) | 62/100 (62) | 0.87 [0.67-1.12] | |  |
| Pulmonary Tuberculosis |  |  |  | |  |
| Yes | 18 (10.0) | 6/18 (33.3) |  | | 0.023* |
| No | 162 (90.0) | 99/162 (61.1) | 0.55 [0.28-1.06] | |  |
| Herpes Zoster |  |  |  | |  |
| Yes | 31 (17.2) | 22/31 (71) |  | | 0.117 |
| No | 149 (82.8) | 83/149 (55.7) | 1.27 [0.98-1.66] | |  |
| Candidiasis |  |  |  | |  |
| Yes | 23 (12.8) | 14/23 (60.9) |  | | 0.792 |
| No | 157 (87.2) | 91/157 (58) | 1.05 [0.74-1.50] | |  |
| Criptococosis |  |  |  | |  |
| Yes | 13 (7.2) | 6/13 (46.2) |  | | 0.355 |
| No | 167 (92.8) | 99/167 (59.3) | 0.78 [0.43-1.42] | |  |
| Coccidiosis |  |  |  | |  |
| Si | 9 (5.0) | 2/9 (22.2) |  | | 0.564 |
| No | 171 (95.0) | 103/171 (60.2) | 0.37 [0.11-1.26] | |  |
| Sexual Transmitted Infection |  |  |  | |  |
| Syphilis | 23 (12.8) | 10/23 (43.5) |  | | 0.053 |
| Condylomas | 9 (5.0) | 3/9 (33.3) | 0.77 [0.27-2.16] | |  |
| Other | 22 (12.2) | 17/22 (77.3) | 1.78 [1.06-2.98] | |  |
| None | 126 (70) | 75/126 (59.5) | 1.37 [0.84-2.23] | |  |
| Side effects with actual ART prescription | | | | |  |
| Yes | 92 (51.1) | 62/92 (67.4) |  | | 0.012* |
| No | 88 (48.9) | 43/88 (48.9) | 1.38 [1.07-1.78] | |  |
| Night sweating |  |  |  | |  |
| Yes | 12 (6.7) | 8/12 (66.7) |  | | 0.545 |
| No | 168 (93.3) | 97/168 (57.7) | 1.15 [0.76-1.76] | |  |
| Insomnia |  |  |  | |  |
| Yes | 11 (6.1) | 5/11 (45.5) |  | | 0.563 |
| No | 169 (93.9) | 100/169 (59.2) | 0.77 [0.40-1.49] | |  |
| Vomit |  |  |  | |  |
| Yes | 11 (6.1) | 8/11 (72.7) |  | | 0.494 |
| No | 169 (93.9) | 97/169 (57.4) | 1.27 [0.86-1.86] | |  |
| Muscular pain |  |  |  | |  |
| Yes | 10 (5.6) | 5/10 (50) |  | | 0.826 |
| No | 170 (94.4) | 100/170 (58.8) | 0.85 [0.45-1.60] | |  |
| Heartburn / stomach pain |  |  |  | |  |
| Yes | 24 (13.3) | 17/24 (70.8) |  | | 0.182 |
| No | 156 (86.7) | 88/156 (56.4) | 1.26 [0.94-1.68] | |  |
| Skin rash |  |  |  | |  |
| Yes | 21 (11.7) | 13/21 (61.9) |  | | 0.724 |
| No | 159 (88.3) | 92/159 (57.9) | 1.07 [0.75-1.53] | |  |
| Nausea |  |  |  | |  |
| Yes | 19 (10.6) | 13/19 (68.4) |  | | 0.346 |
| No | 161 (89.4) | 92/161 (57.1) | 1.20 [0.86-1.67] | |  |
| Headache |  |  |  | |  |
| Yes | 21 (11.7) | 16/21 (76.2) |  | | 0.077 |
| No | 159 (88.3) | 89/159 (56) | 1.36 [1.03-1.79] | |  |
| Action taken with side effects | | | | |  |
| Ask for medical advice | 57 (62.0) | 38/57 (66.7) |  | | 0.031* |
| Wait it to stop | 28 (30.4) | 22/28 (78.6) | 1.18 [0.90-1.54] | |  |
| Keep taking my ART | 4 (4.3) | 2/4 (50) | 0.75 [0.28-2.03] | |  |
| Did not answer | 3 (3.3) | 0/3 (0) | - | |  |
| ART abandoned more than 30 days within the last year | | | | |  |
| Yes | 9 (5) | 7/9 (77.8) |  | | 0.386 |
| No | 171 (95) | 98/171 (57.3) | 1.36 [0.94-1.97] | |  |
| ART abandoned at anytime |  |  |  | |  |
| Yes | 83 (46.1) | 72/83 (86.7) |  | | 0.001* |
| No | 97 (53.9) | 33/97 (34) | 2.55 [1.91-3.41] | |  |
| Length of ART abandon |  |  |  | |  |
| > 30 days | 15 (18.1) | 9/15 (60) |  | | 0.001* |
| 10 - 30 days | 16 (19.3) | 14/16 (87.5) | 1.46 [0.93-2.29] | |  |
| 1 - 9 days | 52 (62.7) | 49/52 (94.2) | 1.57 [1.03-2.39] | |  |
| Reason for abandonment |  |  |  | |  |
| Forgetfulness or neglect when attending a commitment, meeting or work | 35 (40.7) | 35/35 (100) |  | | 0.033* |
| Bureaucracy to get access to ART | 17 (19.8) | 13/17 (76.5) | 0.76 [0.59-1.00] | |  |
| Lack of privacy or feeling better | 11 (12.8) | 9/11 (81.8) | 0.82 [0.62-1.08] | |  |
| Fear of side effects | 8 (9.3) | 6/8 (75) | 0.75 [0.50-1.12] | |  |
| Others | 15 (17.4) | 11/15 (73.3) | 0.73 [0.54-1.00] | |  |
| Drug abuse |  |  |  | |  |
| Alcohol | 40 (22.2) | 23/40 (57.5) |  | | 0.556 |
| Tobacco and Alcohol | 16 (8.9) | 12/16 (75) | 1.30 [0.88-1.92] | |  |
| Others | 4 (2.2) | 2/4 (50) | 0.87 [0.32-2.40] | |  |
| None | 120 (66.7) | 68/120 (56.7) | 0.99 [0.72-1.34] | |  |
| Tobacco |  |  |  | |  |
| Weekly | 8 (4.4) | 5/8 (62.5) |  | | 0.354 |
| Monthly | 10 (5.6) | 8/10 (80) | 1.28 [0.69-2.38] | |  |
| None | 162 (90) | 92/162 (56.8) | 0.91 [0.52-1.58] | |  |
| Alcohol |  |  |  | |  |
| Weekly | 10 (5.6) | 7/10 (70) |  | | 0.368 |
| Monthly | 46 (25.6) | 28/46 (60.9) | 0.87 [0.55-1.39] | |  |
| None | 124 (68.9) | 70/124 (56.5) | 0.81 [0.52-1.24] | |  |
| I have disclosed my family my HIV diagnosis | | | | |  |
| Yes | 143 (79.4) | 84/143 (58.7) |  | | 0.435 |
| No | 35 (19.4) | 19/35 (54.3) | 0.92 [0.66-1.29] | |  |
| Did not to answer | 2 (1.1) | 2/2 (100) | 1.70 [1.48-1.95] | |  |
| I have disclosed my friends my HIV diagnosis | | | | |  |
| Yes | 66 (36.7) | 44/66 (66.7) |  | | 0.146 |
| No | 113 (62.8) | 60/113 (53.1) | 0.80 [0.62-1.02] | |  |
| Did not to answer | 1 (0.6) | 1/1 (100) | 1.50 [1.26-1.78] | |  |
| I have disclosed my HIV diagnosis at workplace | | | | |  |
| Yes | 15 (8.3) | 9/15 (60) |  | | 0.937 |
| No | 161 (89.4) | 94/161 (58.4) | 0.97 [0.63-1.50] | |  |
| Did not to answer | 4 (2.2) | 2/4 (50) | 0.83 [0.29-2.41] | |  |
| Satisfaction with physician |  |  |  | |  |
| Not satisfied | 4 (2.2) | 2/4 (50) |  | | 0.652 |
| Neither unsatisfied nor satisfied | 14 (7.8) | 10/14 (71.4) | 1.43 [0.51-4.02] | |  |
| Satisfied | 162 (90.0) | 93/162 (57.4) | 1.15 [0.43-3.09] | |  |
| Satisfaction with nurses |  |  |  | |  |
| Not satisfied | 1 (0.6) | 1/1 (100) |  | | 0.070 |
| Neither unsatisfied nor satisfied | 4 (2.2) | 4/4 (100) | - | |  |
| Satisfied | 175 (97.2) | 100/175 (57.1) | 0.57 [0.50-0.65] | |  |
| *PR= prevalence ratio*  ** p-value <0.05 ** Mean [IQR]* | | | | | |

*Zidovudine = ZDV; Lamivudine = 3TC; Efavirenz = EFV; Tenofovir = TDF; Abacavir = ABC; Atazanavir = ATV; Ritonavir = RTV; Lopinavir = LPV*
